# Supplementary material for: All-Trans Retinoic Acid Increases DRP1 Levels and Promotes Mitochondrial Fission
Source: Cells. 2021 May 14;10(5):1202. doi: 10.3390/cells10051202 (PMC8156392; doi:10.3390/cells10051202)
Supplement: Supplementary file 1 [file cells-10-01202-s001.zip › cells-1203298-supplementary.pdf]

Supplementary Materials

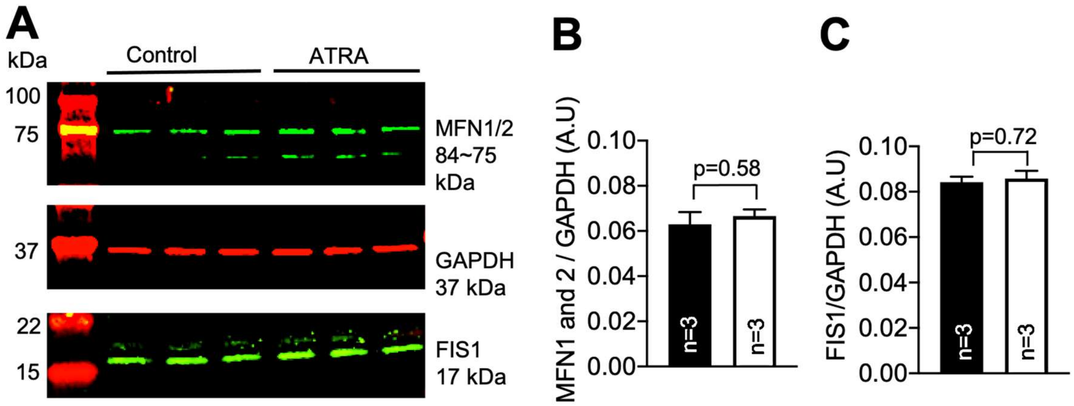

Figure S1. MFN1/2 and FIS1 levels in HEK293 cells treated with ATRA or DMSO (vehicle control). (**A**) Western blots of MFN1/2 (upper panel), GAPDH (middle panel) and FIS1 (lower panel). (**B** and **C**) There was no effect of ATRA on MFN1/2 (B, $p = 0.58$, unpaired 2-sample t-test) and FIS1 (C, $p = 0.72$, vs. control; unpaired 2-sample t-test).

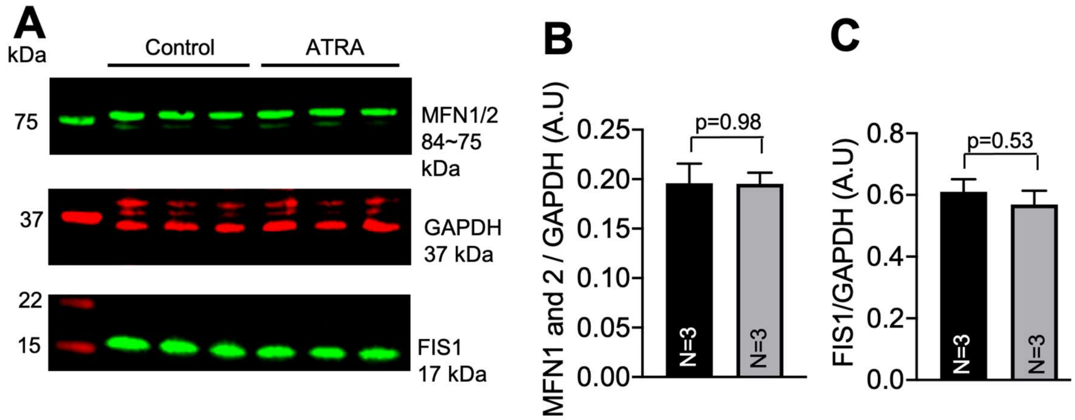

Figure S2. MFN1/2 and FIS1 levels in mouse hearts treated with ATRA or corn oil (vehicle control) via i.p. injection. (**A**) Western blots of MFN1/2 (upper panel), GAPDH (middle panel) and FIS1 (lower panel). (**B**) There was no effect of ATRA on MFN1/2 ($p = 0.98$ vs. control; unpaired 2-sample t-test) and (**C**) FIS1 ($p = 0.53$ vs. control; unpaired 2-sample t-test).
